# Gestational diabetes mellitus and its associated factors in Ethiopia: a systematic review and meta-analysis

**DOI:** 10.1186/s40001-023-01088-5

**Published:** 2023-03-15

**Authors:** Fentahun Yenealem Beyene, Bekalu Getnet Kassa, Gedefaye Nibret Mihretie, Alemu Degu Ayele

**Affiliations:** 1 Department of Midwifery, College of Medicine and Health Science, Bahir Dar University, P.O. Box 79, Bahir Dar, Ethiopia; 2 Department of Midwifery, College of Medicine and Health Sciences, Debre Tabor University, Bahir Dar, Amhara, Ethiopia

**Keywords:** Gestational, Diabetes mellitus, Systematic review, Meta-analysis, Ethiopia

## Abstract

**Background:**

In Ethiopia, gestational diabetes mellitus (GDM) is a significant public health issue and a risk to maternal and child health. Understanding the prevalence and factors of GDM in Ethiopia may also help determine the best interventions. Therefore, we tried to review gestational diabetes and its factors in Ethiopia.AQ: Please check and confirm the edit made to the article title.yes i have checked and confirm

**Methods:**

The Preferred Reporting Items for Systematic Reviews and Meta-Analyses (PRISMA) instrument was used to conduct the review. In order to report on the prevalence and contributing factors of gestational diabetes mellitus, the following databases were used: Google Scholar, PubMed, EMBASE, Scopus, Web of Sciences, and Grey literature. Pilo-tests were conducted using a standardized data gathering form in research using a random sample. All statistical analyses were performed using STATA version 16 software for Windows and the random-effects meta-analysis method. The results are presented using texts, tables, and forest plots, along with measure of effect and a 95% confidence interval.Affiliations: Please confirm if the author names are presented accurately and in the correct sequence (given name, middle name/initial, family name). Author Given name: [Fentahun Yenealem], Last name [Beyene], Given name: [Bekalu Getnet], Last name [Kassa], Given name: [Gedefaye Nibret], Last name [Mihretie], Given name: [Alemu Degu], Last name [Ayele].yes checked and corrected AQ: Is this word Pilo-tests spelled correctly throughout the article?Thank you the correction Affiliations: Please check and confirm whether the city name is correctly identified for the affiliation 2.yes checked and corrected

**Results:**

Out of 1755 records, 10 studies with 6525 participants that fully satisfy the inclusion criteria were included for the meta-analysis. The pooled prevalence of gestational diabetes mellitus in Ethiopia was 12.04% [95% CI (8.17%, 15.90%)]. Inadequate dietary diversity, high body mass index, having a family history of DM, history of having macrosomic neonate, low physical activity, and previous history of GDM were statistically significant.AQ: Please note that the sentence Inadequate dietary diversity, high body mass index… is repeated under the below heading Conclusion.yes checked and corrected

**Conclusion:**

The pooled prevalence of gestational diabetes mellitus is high in Ethiopia. Inadequate dietary diversity, high body mass index, having a family history of DM, history of having macrosomic neonate, low physical activity and previous history of GDM were statically significant variables. Emphasize on early screening, prenatal care and all women having risk factors and trying to get pregnant should get screens for diabetes to improve the maternal and child health at large.AQ: Please check the clarity of the sentence Emphasize on early screening, prenatal…it is clear and easly understand the readers

**Supplementary Information:**

The online version contains supplementary material available at 10.1186/s40001-023-01088-5.

## Introduction

Diabetes is a chronic condition that develops when either the pancreas does not create enough insulin or when the body does not utilize the insulin that is produced properly. It classified as Pre-gestational Diabetes Mellitus (Type I or Type II) and Gestational Diabetes Mellitus (GDM) [1]. One of the most prevalent endocrinopathies and medical consequences of pregnancy is gestational diabetes mellitus (GDM), which is purportedly brought on by pregnancy due to heightened physiological changes in glucose metabolism, placental synthesis diabetogenic substance, and maternal insulin resistance [2–8].Article structure: Please check and confirm the section headings are correctly identified.yes checked and corrected 

It is defined as the initial diagnosis or recognition of glucose intolerance during pregnancy and does not meet the criteria for overt diabetes outside of pregnancy [4, 9–12].

It is linked to serious short- and long-term morbidities for both the mother and the fetus (fetal morbidities include spontaneous abortion, preterm birth, malformations, altered fetal growth, unexplained fetal demise, respiratory distress syndrome, hydraminous, hypoglycemia and hypocalcaemia, hyperbilirubinemia, polycythemia, cardiomyopathy, and long-term cognitive development delay; and maternal morbidities includes Preeclampsia, Diabetic Nephropathy, Diabetic Retinopathy, Diabetic Neuropathy, Diabetic Ketoacidosis and infections [12–23].

The pooled global overall prevalence of GDM, irrespective of the screening threshold categories, was 4.4% [24]. And in Africa was 13.61% and 14.28% in the sub-Saharan African region [25].

Risk factors of GDM include excessive body weight, low level of physical activity, consanguineous marriage, previous history of GDM, glycated hemoglobin > 5.7%, history of cardiovascular disease, overweight and obese reproductive-age females soars, previous history of spontaneous abortion, age, parity, antenatal depression, family history of type 2 DM, having previous macrosomic baby, and a history of still birth [26–32].

The 2022 Standards of Care gives more emphasis on diabetes screening for women who are trying to get pregnant or are already pregnant. If they have risk factors, women who are trying to get pregnant should get screens for diabetes. Additionally, healthcare professionals should consider about screening all women who intend to get pregnant for diabetes who have not yet been diagnosed. Similar to this, screenings for pregnant women at risk should begin before week 15 of the pregnancy or in the initial prenatal appointment [33]. It is challenging to assess the prevalence of GDM between and within nations due to the lack of consistency in the diagnostic protocols, which vary not only between countries but also within countries. However, in 2013, taking into account the concerns raised by the International Association of Diabetes in Pregnancy Study Groups (IADPSG) recommendations, WHO changed its criteria for the diagnosis of GDM [34–36]. Ethiopia endorses the 2013 WHO screening and diagnosis standards and GDM may be diagnosed based on the fasting plasma glucose that is 92–125 mg/dL (5.1–6.9 mmol/L) and/after a 75 g oral glucose load, plasma glucose is 180 mg/dL (10.0 mmol/L) and/after a 75 g oral glucose load, plasma glucose is 153–199 mg/dL (8.5–11.0 mmol/L) at two hours [9].

There has not been a comprehensive study of the prevalence of GDM at the national level, only small-scale research at different regional and zonal levels. Therefore, the purpose of this meta-analysis is to estimate the prevalence of GDM and its contributing factors at the national level in a more comprehensive manner. The results of this study would emphasize the significance and urgency of expanding GDM screening and its care throughout Ethiopia. Understanding the prevalence and factors of GDM in Ethiopia may also help determine the best intervention to use in order to lessen the severity of the issue, enhance mother and child health, and end the burden of GDM in Ethiopia. As a result, we conducted a systematic review and meta-analysis to assess the prevalence and to determine GDM in Ethiopia.

## Methods

### Design and search strategy

This study followed a predetermined protocol and examined the data to determine the prevalence of gestational diabetes mellitus and its contributing factors in Ethiopia. We used the Preferred Reporting Items for Systematic Reviews and Meta-Analyses (PRISMA) criteria to review and present the findings of this systematic review and meta-analysis [37] in detail (Additional file 1).

For reporting the prevalence of gestational diabetes mellitus and its factors, a systematic and thorough literature search methodology was used without restriction from the beginning of the study until sending to the journal; via Google Scholar, PubMed, EMBASE, Scopus, Web of Sciences, and Grey literature databases. To find more pertinent articles, we manually looked for cross-references as well. The terms “prevalence of gestational diabetes mellitus,” “gestational diabetes mellitus,” “associated factors of gestational diabetes mellitus,” “diabetes mellitus,” “complication of gestational diabetes mellitus,” screening and diagnosis of gestational diabetes mellitus, and “Ethiopia” have all been included in our search strategy.

### Inclusion and exclusion criteria

#### Inclusion criteria

This review included all peer-reviewed, published, and repository research that addressed the prevalence of gestational diabetes mellitus, its risk variables, and reported accurate measures of relationship.

#### Exclusion criteria

Reviews, case studies, conference abstracts, letters, studies in which the proper measures of association were not presented, abstracts without additional details or without full-text articles and duplicate data were excluded.

#### Data collection and synthesis

Based on the inclusion and exclusion criteria, two independent authors (FYB and BGK) evaluated the eligibility of all retrieved papers by looking at the title and abstract. Additionally, three authors (FYB, GNM, and ADA) evaluated the studies' quality. We came to a rational consensus when there was a disagreement.

#### Data extraction

Pilo-tests were conducted using a standardized data gathering form in research using a random sample. First author, publication year, data collection year, study setting, study design, sample size, response rate, methods of diagnosis, statistically significant factors, adjusted Odds ratio (AOR), 95% confidence interval, prevalence, and covariance are all taken into account in the data collection. When results were published more than once, the data were only taken into account once. A logical agreement between the two authors was able to clear up any doubts that arose during the extraction procedure. We have excluded the research or the unavailable parameter in the event of incomplete data.

#### Study quality and risk of bias assessment

The quality and strength of each study are to be evaluated using the development of a critical assessment instrument for use in systematic reviews addressing problems of prevalence and incidence [38] in detail (Additional file 2). We evaluated based on the following assessment criteria: inclusion/exclusion criteria, sampling procedure, sample size, sample representativeness, data collection techniques, and adequate response rate (Table 1).

**Table 1:** Characteristics of studies which are included in the systematic review and meta-analysis, 2022

References	Study year	Region	Study area	Study design	Sample size	Prevalence of gestational diabetes
Larebo et al. [42]	2020	SNNPR	Hadiya zone	Cross-sectional	470	26.2
Ewnetu et al. [48]	2016	Adiss Ababa	Adiss Ababa	Cross-sectional	162	29.6
Nigatu et al. [47]	2017	Adiss Ababa	Adiss Ababa	Cross-sectional	422	16.9
Seyoum et al. [50]	1999	Tigray	Tigray	Cross-sectional	890	3.7
Dedecha et al. [46]	2021	Oromia	Guji	Cross-sectional	190	7.4
Muche et al. [49]	2018	Amhara	Northwest Ethiopia	Cross-sectional	1110	12.8
Woticha et al. [44]	2017	SNNPR	Wolaita	Cross-sectional	564	4.2
Atlaw et al. [45]	2020	Oromia	Goba town	Prospective cohort	500	15.7
Wakwoya et al. [51]	2017	Harar	Harar and Dire Dawa	Case–control	1834	2.6
Boda et al. [43]	2019	SNNPR	Gamo zone	Cross-sectional	383	7.1

#### Data synthesis and statistical analysis

A meta-analysis was carried out to provide a comparative classification of the outcome and determinants of interest for the selected publications and to calculate the effect size for the prevalence of gestational diabetes mellitus in Ethiopia. The related factors of gestational diabetes mellitus were examined based on eligibility requirements. With regard to one linked factor of gestational diabetes mellitus, at least two studies were taken into consideration, together with their respective measures of effect and 95% confidence intervals (CI). Calculating the effect size and 95% confidence interval provided an approximation of the substantial relationship between gestational diabetes mellitus and its contributing components (CI). A DerSimonian–Laird method-based random effects model was taken into consideration in order to identify variations both within and between studies [39]. In addition, *I*^2^ statistics and Cochran's *Q* test have been used to measure heterogeneity through studies. The percentage of the sample's overall variance that can be attributed to heterogeneity is thought to be measured by the *I*^2^ statistics. *I*^2^ values range from 0 to 100%, with *I*^2^ ≥ 75% signifying significant study heterogeneity [39]. We looked at publication bias qualitatively in the meta-analysis with funnel plot and used Begg's test and Egger's test (*P* 0.05) to determine statistical significance [41]. STATA version 16 was used for the statistical analysis. The results are provided using texts, tables, and forest plots with measures of effect and 95% confidence interval.

### Sensitivity and subgroup analysis

Using sensitivity analysis of the chosen studies, we investigate potential sources of heterogeneity. Sensitivity analysis was used to assess the impact of inappropriate studies. A subgroup analysis was also performed for prevalence by place of study, region, and sample size.

### Operational definitions

Gestational diabetes: It is defined as the initial diagnosis or recognition of glucose intolerance during pregnancy. It was diagnosed using the one-step strategy by performing a 75-g oral glucose tolerance test (OGTT) protocol, with plasma glucose measurement taken when patient is fasting and at 1 and 2 h, at 24–28 weeks of gestation in women not previously diagnosed with overt diabetes. The OGTT was performed in the morning after an overnight fast of at least 8 h [2, 4, 9].

## Results

**Fig. 1:**
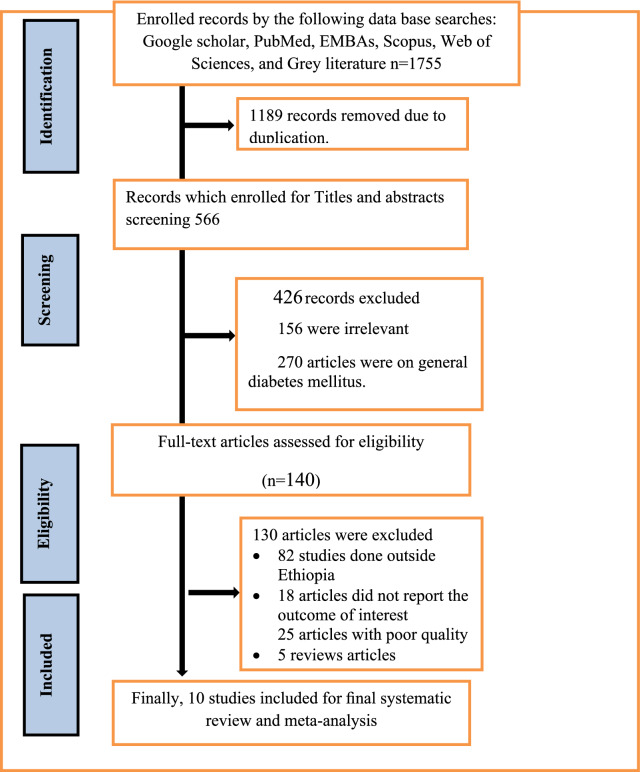
PIRSMA flowchart diagram of the study selection

A total of 1755 research were reviewed; of these, 426 studies were removed because the information in the title and abstract did not line up, and also 1189 papers were removed due to duplication. A thorough evaluation of the text led to the removal of another130 articles from the review due to duplication, incorrect statistical analysis, conflicting results publication, inconsistent research outcome, or irrelevant target participants. Finally, ten studies were taken into account for the pooled estimation of gestational diabetes mellitus and its factor analysis (Fig. 1). Among the included studies, nine of them were published articles while one of them was repository articles. Eight of the included studies were cross-sectional in design, the remaining two were cohort and case–control studies, and around 90% of the study setting of the included studies was institutional (Table 1).

### Characteristics of the included studies

A total of 10 studies with 6525 participants were considered. Of those, three studies [42–44] were conducted in SNNPR, two [45, 46] in Oromia region, two [47, 48] in Addis Ababa city administration, and the rest three [49–51] in other regions (Amhara, Tigray, and Harari), respectively.

### Prevalence of gestational diabetes mellitus

**Fig. 2:**
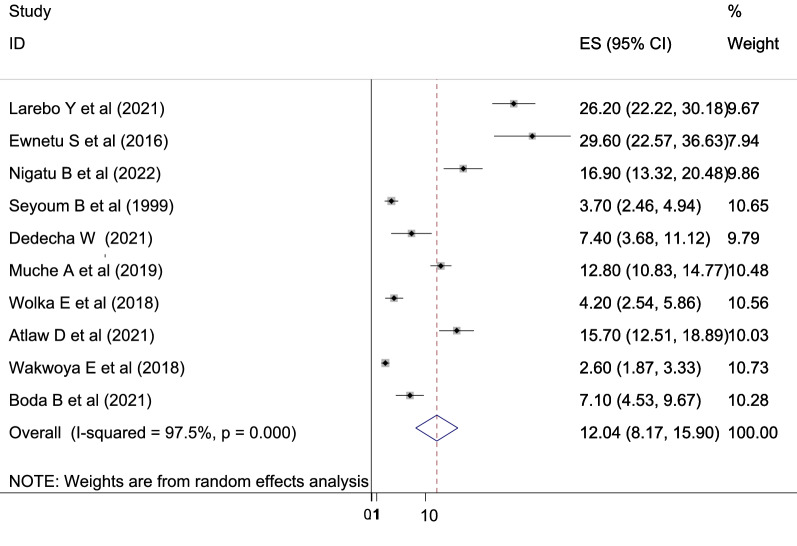
Forest plot for pooled prevalence of gestational diabetes mellitus

In Ethiopia, the pooled estimate of gestational diabetes mellitus using a random effects model was 12.04% (95% CI 8.17%, 15.90%) with significant heterogeneity between studies (*I*^2^ = 97.5, *P* = 0.000) (Fig. 2).

**Fig. 3:**
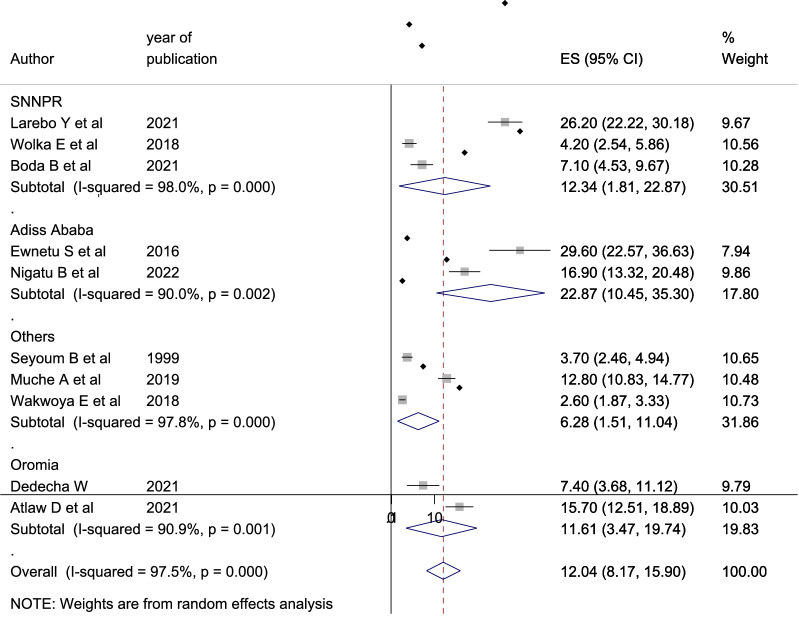
Subgroup analysis by region on gestational diabetes mellitus

According to the subgroup analysis by region, Addis Ababa has the highest rate of gestational diabetes mellitus [12.343% (95% CI 1.814, 22.873), *I*^2^ = 98%], and category of in other regions (Tigray, Amhara and Harari) region has the lowest rate [6.28 (95% CI 1.51, 11.04), *I*^2^ = 97.8%] (Fig. 3).

AQ: Consider rephrasing the given sentence According to the subgroup analysis by region, Addis…thank you for your correction

### Checking for heterogeneity and publication bias

**Fig. 4:**
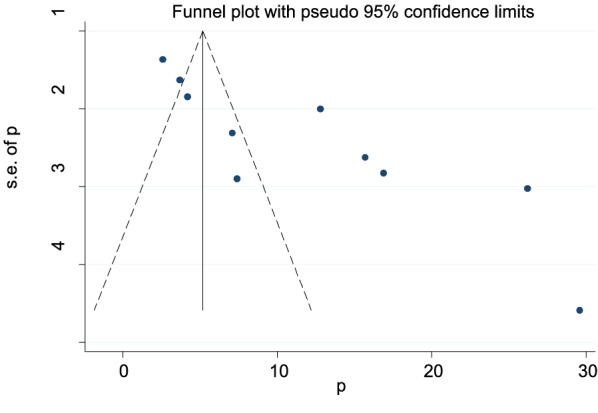
Funnel plot to show the publication bias in 10 studies

**Fig. 5:**
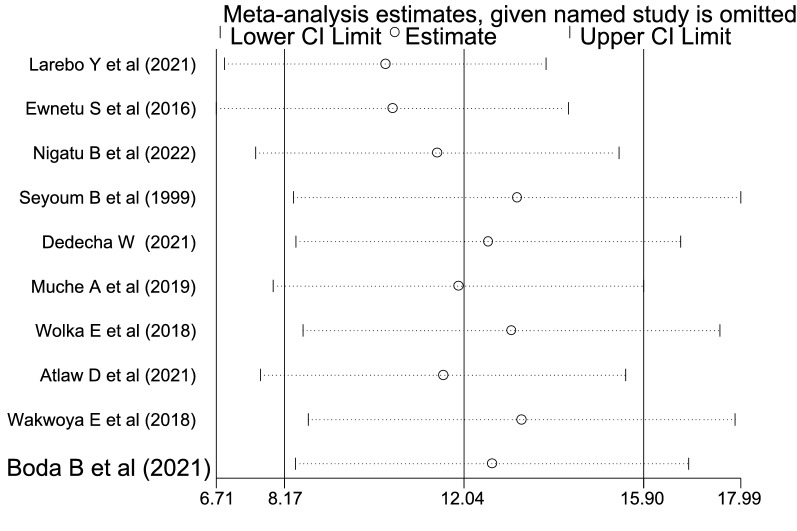
Sensitivity analysis of the 10 studies

The overall meta-analysis result suggests that there was statistically substantial heterogeneity among studies (*I*^2^ = 97.5%), and we attempted to perform a subgroup analysis by region to reduce and modify heterogeneity (Fig. 3). Using the funnel plot and Egger's test to analyze the publication bias, it was determined that the included studies were distributed asymmetrically (Fig. 4). For the existence of publication bias, the Egger's test result was statistically significant (*P* = 0.0001). Additionally, we attempted to do the sensitivity analysis using the random-effects model and proposed that none of the studies had an impact on the total estimate (Fig. 5).

### Factors associated with gestational diabetes mellitus (GDM)

**Fig. 6:**
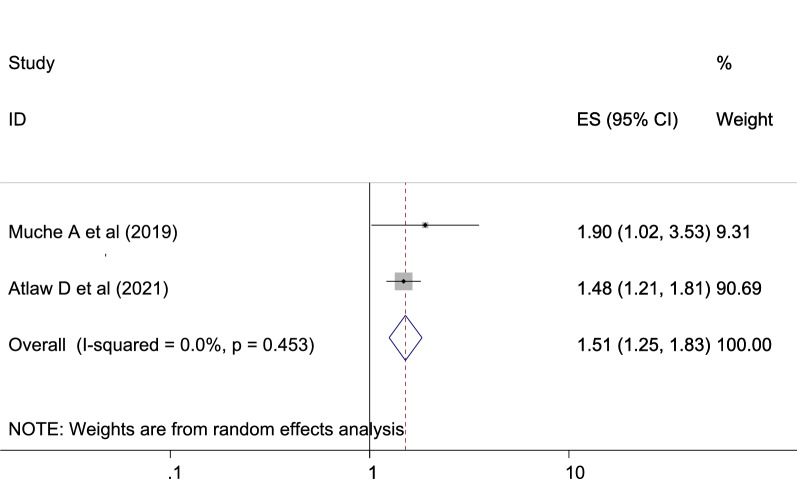
Forest plot showing the association between gestational diabetes mellitus and inadequate dietary intake

**Fig. 7:**
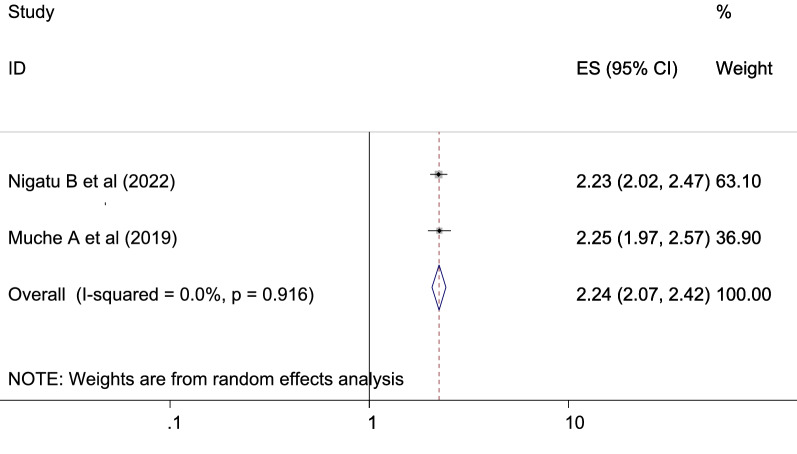
Showing that the association of gestational diabetes mellitus and body mass indexAQ: Should the caption Showing that the association of be changed to Forest plot showing the association of… in Figures 7 to 11 captions? thank you your recommendation and accepted

**Fig. 8:**
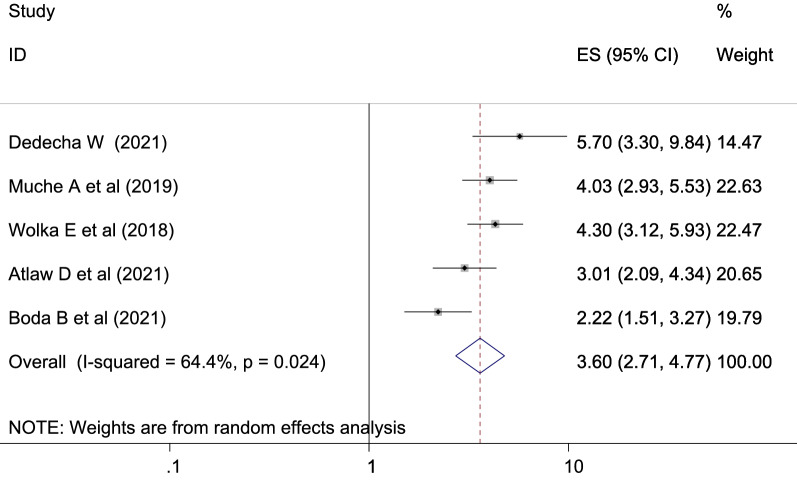
Showing the association between gestational diabetes mellitus and family history of diabetes mellitus

The reviewed literature showed various significant factors associated with gestational diabetes mellitus in Ethiopia. Inadequate dietary intake were more risks to gestational diabetes mellitus than the counter parts which is adequate dietary intake with [OR: 1.51 (95% CI (1.25, 1.83), *I*^2^: 0.0%] (Fig. 6). The heterogeneity test (*P* = 0.453) showed no evidence of variation across studies. The result of Egger’s test showed no statistically significant publication bias (*P* = 0.25). The odds of developing GDM among pregnant women with BMI of ≥ 25 kg/m^2^ were more likely than those with a BMI of < 25 kg/m^2^ which is statistically significant [OR: 2.24 (2.07, 2.42), *I*^2^: 0.0%] (Fig. 7). The heterogeneity test (*P* = 0.916)] showed that there is no significant variation across studies. The result of Egger’s test showed that there is no statically significant evidence of publication bias (*P* = 0.06). Participants who had a family history of diabetes mellitus were a higher chance of developing gestational diabetes mellitus as compared to those who had no family history of diabetes mellitus which is statistically significant [OR: 3.60 (2.71, 4.77), *I*^2^: 64.4%] (Fig. 8). The heterogeneity test (*P* = 0.024) showed that there is no significant variation across studies. The result of Egger’s test showed no statistically significant evidence of publication bias (*P* = 0.145).

AQ: Please check the clarity of the given sentence Inadequate dietary intake were more risks to gestational diabetes…yes checked and corrected 

**Fig. 9:**
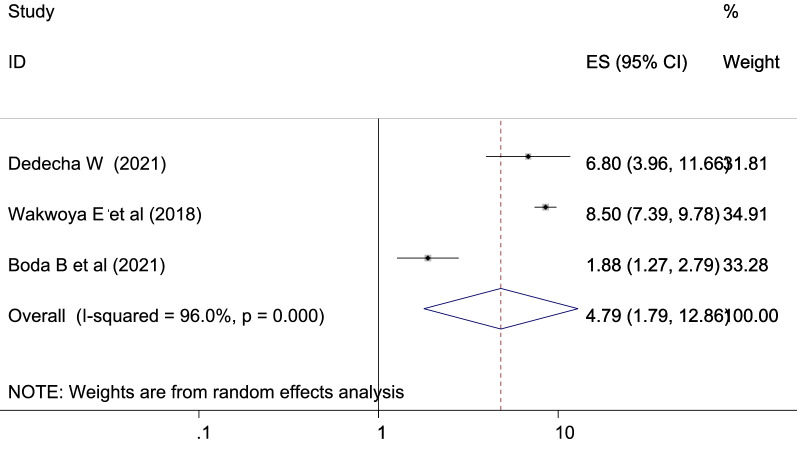
Showing the relation between gestational diabetes mellitus and previous macrosomic baby

Pregnant mother who had macrocosmic baby (> 4 kg) was more likely to develop GDM as compared to pregnant mother who had no macrocosmic baby previously which is statistically significant [OR: 4.79 (1.79, 12.86), *I*^2^: 96.0%] (Fig. 9). The heterogeneity test (*P* = 0.001) showed no significant variation across studies. The result of Egger’s test showed no statistically significant evidence of publication bias (*P* = 0.431).

**Fig. 10:**
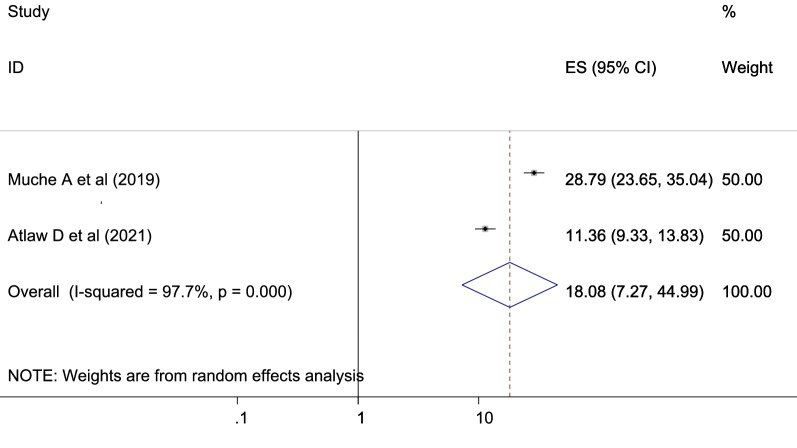
Showing that the association of gestational diabetes mellitus and low physical activities

Pregnant women with low physical activity were more at risk of GDM than those with high physical activity which is statistically significant [OR 18.08 (7.27, 44.99), *I*^2^ 97.7%] (Fig. 10). The heterogeneity test (*P* = 0.001 showed no variation across studies. The result of Egger’s test showed no statistically significant evidence of publication bias (*P* = 0.743).

**Fig. 11:**
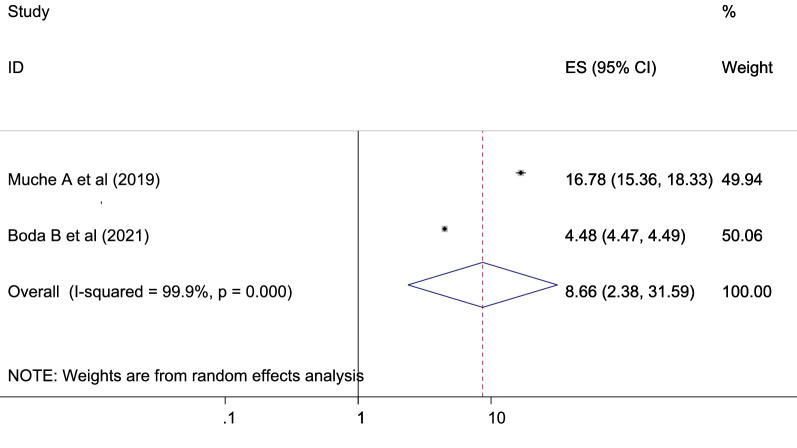
Showing that the association of gestational diabetes mellitus and previous history of gestational diabetes

Women with a previous history of GDM had higher chance of developing GDM as compared to women without a history of GDM which is statistically significant [OR 8.66 (2.38, 31.59), *I*^2^ 99.9%] (Fig. 11). The heterogeneity test (*P* = 0.001) showed no variation across studies. The result of Egger’s test showed no statistically significant evidence of publication bias (*P* = 0.523).

## Discussion

The increased prevalence of gestational diabetes mellitus around the world has led to new findings about the link between blood sugar levels and the success of pregnancies [52].

This review showed that the pooled prevalence of gestational diabetes mellitus was 12.04% (95% CI 8.17%, 15.90%) in Ethiopia. This finding was in line with studies conducted in Africa: systematic review and meta-analysis (13.61%) [25], in Libreville (10.2%) [53], Itojo General Hospital, South Western Uganda (15.6%) [54], in Asia: a systematic review (11.5%) [55], eastern and southeastern Asia (10.1%) [56], Europe Systematic Review (10.9%) [57], and Kumasi, Ghana (8.5%) [58]. This might be due to using related screening approach and definitions.

However, the findings of this review were lower than the studies conducted in Bangladesh demographic finding (35%) [59], Lima, Peru (16%) [60], Punjab, North India (35%) [61], Dodoma region, Tanzania (27.5%) [62], Riyadh, Saudi Arabia (32.6%) [63], and in Limbe, Cameroon (20.5%) [64]. The variation might be due to variation in different diagnostic approach, operational definitions, study population, areas and methods used.

On the contrary, the pooled prevalence of gestational diabetes mellitus was higher than the studies conducted in sub-Saharan Africa: systematic review (2–6%) [65], Nigeria (2.98%) [66], western Kenya (2.9%) [67], Kigali City, Rwanda (8.3%) [68], Europe: a meta-analysis (5.4%) [69], in the National Health and Nutrition Examination Surveys in USA, 2007–2014 (7.6%) [70], southern Tanzania (4.3%) [71], Yemen (5.1%) [72], Turkey: systematic review (7.7%) [73], and Brazilian Public Health Care (5.4%) [74]. This might be due to study year variation, socio-demographic characteristics of the study participants, sample size, measurement tools used, and variation in diagnostic approach and variation in operational definitions.

The subgroup analysis by region showed that the highest level of GDM was in Addis Ababa which is 22.87% whereas the lowest was in other three regions (Tigray, Amhara, and Harar) which is 6.28%. This might be because women living at the federal level have more awareness about timing, benefit, and early screening of GDM than those living at the regional level.

According to our review and meta-analysis finding, inadequate dietary diversity, high body mass index, having family history of DM, history of having macrosomic neonate, low physical activity, and previous history of GDM were the statically significant variables.

Women who get inadequate dietary diversity were two times more exposed to GDM than the counters with OR: 1.51 [95% CI (1.25, 1.83)]. This finding is in agreement with the studies conducted in coastal Karnataka [75]. Inadequate dietary diversity may result in a reduced likelihood of obtaining various vitamins, minerals, nutrients, and phytochemicals that can help prevent nutrient deficiencies and chronic diseases. It may also increase stress, fatigue, and less capacity for work. Over time, it may also increase the risk of contracting certain diseases and other health issues, such as being overweight or obese and having an abnormal metabolism.

Participants with body mass index > 30 were high chance of getting GDM compared with normal level of body mass index with OR: 2.24 [95% CI (2.07, 2.42)]. This analysis is similar to the studies in Brazilian Public Health Care [74], southern Tanzania [71], in Riyadh, Saudi Arabia [63], in Limbe, Cameroon [64], Libreville [53], Punjab, North India [61], Lima, Peru [60], and in Asia: a systematic review [55]. Patients with higher BMI gain a higher fat mass accumulation, which could affect subsequent maternal insulin resistance [76]. This may be due to the fact that as a mother's BMI rises, she may engage in less physical activity, experience stress, get weaker, and ultimately develop high fat stores, abnormal metabolism, and GDM.

Women having family history of DM were four times more develop GDM than no family history of DM with OR: 3.60 [95% CI (2.71, 4.77)]. This finding is in agreement with the studies conducted in Fujian province [77], Yemen [72], southern Tanzania [71], in Riyadh, Saudi Arabia [63], Dodoma region, Tanzania [62], Lima, Peru [60], Asia: a systematic review [55], and in coastal Karnataka [75]. This may be connected to pregnant women with a family history of diabetes and having a genetic predisposition that could lead to the development of GDM.AQ: Consider rephrasing the given sentence Women having family history of DM were…yes checked and no need of rephrasing

Women with a history of macrosomic neonate were more prevalent to GDM than the counter parts with OR: 4.79 [95% CI (1.79, 12.86)]. This is in agreement with the studies in southern Tanzania [71], in Riyadh, Saudi Arabia [63], in Limbe, Cameroon [64], and in Asia: a systematic review [55]. Previous macrosomic neonates may have been caused by uncontrolled GDM or by hyperglycemia; this may have a substantial correlation with GDM emerging during a subsequent pregnancy.

Participants who had low physical exercise were more prone to GDM than those having high regular exercise with OR: 18.08 [95% CI (7.27, 44.99)]. This finding is in agreement with studies conducted in Dodoma region, Tanzania [71], and in coastal Karnataka [72]. Exercise is deemed to be an important component of lifestyle intervention for GDM [78]. Healthy pregnant women should engage at least 30 min of moderate-intensity exercise at least four times per week [79]. Exercise may be an effective strategy to help control blood sugar levels. If you do not exercise much or at all, your blood sugar levels may be abnormal, which can cause insulin resistance and aberrant metabolism, which can lead to GDM.

Women’s having history of previous GDM were nine times more develop GDM than no history of GDM with 8.66 [95% CI (2.38, 31.59)]. This finding is in agreement with the studies conducted in Yemen [72], in Riyadh, Saudi Arabia [63], and Libreville [53], and in Asia: a systematic review [55]. Approximately, 50–73% of women with previous GDM developed GDM in a future pregnancy [80–82]. Different studies showed that there is a strong association between previous GDM and subsequent pregnancy developing GDM.AQ: Consider rephrasing the given sentence Women’s having history of previous GDM were nine…yes checked and no need of ephrasing

### Limitations

We tried to examine only the influence of six factors because other major factors were not commonly investigated by the included studies. Due to the limitation of published systematic reviews and meta-analysis on gestational diabetes and its factor in national level, it creates difficulty to compare our results with other national evidence.

## Conclusions and recommendations

The pooled prevalence of gestational diabetes mellitus is high in Ethiopia. Inadequate dietary diversity, high body mass index, having family history of DM, history of having macrosomic neonate, low physical activity, and previous history of GDM were statically significant variables. This might be very useful for healthcare policymakers (e.g. Federal Ministry of health, Hospital administrators and NGOs) to emphasize on early screening, prenatal care and all women having risk factors and trying to get pregnant should get screens for diabetes to improve the maternal and child health at large. Given the multifactorial nature of factors influencing gestational diabetes mellitus, further qualitative research is needed to identify additional factors, especially from participants’ perspective, and explore context-specific strategies,AQ: Consider rephrasing the given sentenceThis might be very useful for…yes checked and no need of rephrasing References: As References [25] and [53] are same, we have deleted the duplicate reference and renumbered accordingly. Please check and confirm.yes checked and confirmed

## Supplementary Information

**Additional file 1.** PRISMA-P (preferred reporting items for systematic review and meta-analysis protocols) 2009 checklist: recommended items to address in a systematic review protocol.

**Additional file 2.** Quality assessment of included studies using the Joanna Briggs Institute criteria’s for assessing quality of primary studies and JBI Critical Appraisal Checklist for Studies Reporting Prevalence Data, 2019.

## Data Availability

All the data are available from the corresponding author upon a reasonable request.

## Abbreviations

CI: Confidence interval
DIP: Diabetes in pregnancy
DM: Diabetes mellitus
GDM: Gestational diabetes mellitus
OGTT: Oral glucose tolerance test
PRISMA: Preferred reporting items for systematic reviews and meta-analyses
WHO: World Health Organization

## References

[1] WHO. World Health Organization Department of Non-Communicable Disease Surveillance. Geneva; 1999.

[2] American College of Obstetricians and Gynecologists 409 12th Street SW, Washington, DC 20024-2188; 2022

[3] World Health Organisation. Classification of hyperglycaemia first detected in pregnancy. [Internet]. 2013 [cited 2020 January 30]. Available from: https://www.who.int. 24199271

[4] American College of Obstetricians and Gynecologists: Gestational diabetes mellitus. Practice Bulletin No. 190, February 2018. Reaffirmed 2019a.

[5] Chiefari E, Arcidiacono B, Foti D, Brunetti A (2017). Gestational diabetes mellitus: an updated overview. J Endocrinol Invest.

[6] Lapolla A, Metzger BE (eds): Gestational diabetes. A decade after the HAPO study. In: Front diabetes, vol 28. Basel: Karger; 2020. p 11–20. 10.1159/000480162.

[7] Barbour LA, McCurdy CE, Hernandez TL, Kirwan JP, Catalano PM, Friedman JE (2007). Cellular mechanisms for insulin resistance in normal pregnancy and gestational diabetes. Diabetes Care.

[8] Genevay M, Pontes H, Meda P (2010). Beta cell adaptation in pregnancy: a major difference between humans and rodents?. Diabetologia.

[9] Management protocol on selected obstetrics topics for hospitals. MOH, Ethiopia; 2020.

[10] http://apps.who.int/iris/bitstream/handle/10665/85975/WHO_NMH_MND_13.2_eng.pdf;jsessionid=FD8DC8872A84924274CB92855D70888A?sequence=1. Accessed 12 Oct 2018.

[11] American Diabetes Association (2020). Standards of medical care in diabetes. Diabetes Care.

[12] Nankervis A, McIntyre H, Moses R, Ross G, Callaway L, Porter C, et al. ADIPS consensus guidelines for the testing and diagnosis of gestational diabetes mellitus in Australia and New Zealand. [Internet]. 2014 [cited 2019 November 14]. Available from: https://www.adips.org.

[13] Hod M, Kapur A, McIntyre HD (2019). Evidence in support of the International Association of Diabetes in Pregnancy study groups’ criteria for diagnosing gestational diabetes mellitus worldwide in 2019. Am J Obstet Gynecol.

[14] The HAPO Study Cooperative Research Group (2008). Hyperglycemia and adverse pregnancy outcomes. N Engl J Med.

[15] Brown J, Grzeskowiak L, Williamson K, Downie MR, Crowther CA. Insulin for the treatment of women with gestational diabetes. Cochrane Database of Systematic Reviews. 2017 [cited 2020 January 31]; Issue 11. Art. No.: CD012037 (11). 10.1002/14651858.CD012037.pub2. 10.1002/14651858.CD012037.pub2 PMC6486160 29103210

[16] Tieu J, McPhee AJ, Crowther CA, Middleton P, Shepherd E. Screening for gestational diabetes mellitus based on different risk profiles and settings for improving maternal and infant health. Cochrane Database of Systematic Reviews. [Internet]. 2017 [cited 2020 April 23]; 8 Art No. CD007222. 10.1002/14651858.CD007222.pub4. 10.1002/14651858.CD007222.pub4 PMC6483271 28771289

[17] Lowe WL, Scholtens DM, Lowe LP, Kuang A, Nodzenski M, Talbot O (2018). Association of gestational diabetes with maternal disorders of glucose metabolism and childhood adiposity. J Am Med Assoc.

[18] Berger H, Gagnon R, Sermer M (2019). Guideline no. 393: diabetes in pregnancy. J Obstet Gynaecol Can.

[19] Kong L, Nilsson I, Gissler M, Lavebratt C (2019). Associations of maternal diabetes and body mass index with offspring birth weight and prematurity. J Am Med Assoc Pediatr.

[20] Durnwald A. Gestational diabetes mellitus: glycemic control and maternal prognosis. [Internet]. Waltham, MA: UpToDate Inc; 2020 [cited 2020 July 7]. Available from: https://www.uptodate.com.

[21] Dirar A, Doupis J (2017). Gestational diabetes from A to Z. World J Diabetes.

[22] Battarbee A, Venkatesh K, Aliaga S, Boggess K (2020). The association of pregestational and gestational diabetes with severe neonatal morbidity and mortality. J Perinatol.

[23] Billionnet C, Mitanchez D, Weill A, Nizard J, All F, Hartemann A (2017). Gestational diabetes and adverse perinatal outcomes from 716,152 births in France in 2012. Diabetologia.

[24] Behboudi-Gandevani S, Amiri M, Bidhendi Yarandi R (2019). The impact of diagnostic criteria for gestational diabetes on its prevalence: a systematic review and meta-analysis. Diabetol Metab Syndr.

[25] Muche AA, Olayemi OO, Gete YK (2019). Prevalence and determinants of gestational diabetes mellitus in Africa based on the updated international diagnostic criteria: a systematic review and meta-analysis. Arch Public Health.

[26] Spaight C, Gross J, Horsch A, Puder JJ (2016). Gestational diabetes mellitus. Endocr Dev.

[27] Hod M, Kapur A, Sacks DA, Hadar E, Agarwal M, Di Renzo GC (2015). The international federation of gynecology a\/lmloopi0gioihgftresw2qw7/nd obstetrics (FIGO) initiative on gestational diabetes mellitus: a pragmatic guide for diagnosis, management, and care(). Int J Gynaecol Obstet.

[28] Begum IA (2014). Gestational diabetes mellitus in Primi gravida of Bangladesh in different trimesters. Int J Biol.

[29] Jesmin S, Akter S, Akashi H, Al-Mamun A, Rahman MA, Islam MM, Sohael F, Okazaki O, Moroi M, Kawano S (2014). Screening for gestational diabetes mellitus and its prevalence in Bangladesh. Diabetes Res Clin Pract.

[30] Mannan MA, Rahman MH, Ara I, Afroz H (2012). Prevalence and pregnancy outcome of gestational diabetes mellitus among bangladeshi urban pregnant women. J Med.

[31] Sultana N, Hasanat MA, Jahan S, Hasan M, Aktar Y, Panthi S, Rahman MA, Fariduddin M (2016). Association of risk factors in gestational diabetes mellitus among pregnant mothers attending at a tertiary care hospital in Bangladesh. J Bioinform Diabetes.

[32] Sayeed MA, Jahan S, Rhaman MM, Chowdhury MMH, Khanam PA, Begum T, Ruman U, Banu A, Mahtab H (2013). Prevalence and perinatal outcomes in GDM and non-GDM in a rural pregnancy cohort of Bangladesh. Ibrahim Med Coll J.

[33] ADA Standards of Medical Care in Diabetes—2022. Available https://diabetesjournals.org/care/issue/45/Supplement_1. Accessed Jan. 5, 2022.

[34] World Health Organization (WHO). Diagnostic criteria and classification of hyperglycaemia first detected in pregnancy. 2013. Available from: http://apps.who.int/iris/bitstream/10665/85975/1/WHO_NMH_MND_13.2_eng.pdf. 24199271

[35] Colagiuri S, Falavigna M, Agarwal MM, Boulvain M, Coetzee E, Hod M (2014). Strategies for implementing the WHO diagnostic criteria and classification of hyperglycaemia first detected in pregnancy. Diabetes Res Clin Pract.

[36] Diabetes IAO, Panel PSGC (2010). International association of diabetes and pregnancy study groups recommendations on the diagnosis and classification of hyperglycemia in pregnancy. Diabetes Care.

[37] Moher D, Liberati A, Tetzlaff J, Altman DG, The PRISMA Group (2009). Preferred reporting items for systematic reviews and meta-analyses: the PRISMA statement. PLoS Med.

[38] Munn Z, Moola S, Lisy K, Riitano D, Tufanaru C, Aromataris E, Munn Z (2017). Chapter 5: Systematic reviews of prevalence and incidence. Joanna Briggs Institute reviewer's manual.

[39] Dersimonian R, Laird N (1986). Meta-analysis in clinical trials. Control Clin Trials.

[40] Higgins JP, Thompson SG (2002). Quantifying heterogeneity in a meta-analysis. Stat Med.

[41] Egger M, Davey-Smith G, Altman D (2008). Systematic reviews in health care: meta analysis in context.

[42] Larebo YM, et al. Prevalence and risk factors of gestational diabetes mellitus among women attending antenatal care in Hadiya Zone Public Hospitals, Southern Nation Nationality People Region; Hindawi: BioMed Research International; 2021. 10.1155/2021/5564668 PMC8046536 33880369

[43] Boda B (2021). Assessment of the prevalence of gestational diabetes mellitus and associated factors among women attending antenatal care at Arba Minch Town Public Health Facilities, Southern Ethiopia. OMO Int J Sci.

[44] Woticha EW, Deressa W, Reja A (2018). Prevalence of gestational diabetes mellitus and associated factors in Southern Ethiopia. Asian J Med Sci.

[45] Atlaw D (2021). Incidence and risk factors of gestational diabetes mellitus in Goba town, Southeast Ethiopia: a prospective cohort study. BMJ.

[46] Dedecha W, et al. Prevalence of gestational diabetes mellitus and associated factors among pregnant women following antenatal care at public health facilities in Bule Hora town, Oromia, Southern Ethiopia. http://etd.aau.edu.et/handle/123456789/29144; 2021.

[47] Nigatu B, Workneh T, Mekuria T (2022). Prevalence of Gestational Diabetes Mellitus among pregnant women attending antenatal care clinic of St. Paul’s Hospital Millennium Medical College, Addis Ababa, Ethiopia. Clin Diabetes Endocrinol.

[48] Ewnetu S (2016). Effects of level of glycaemic control in reduction of maternal and perinatal complications among pregnant diabetic women at Tikur Anbessa Specialized Hospital, Addis Ababa, Ethiopia. Ethiop Pharmaceut J.

[49] Muche AA, Olayemi OO, Gete YK (2019). Prevalence of gestational diabetes mellitus and associated factors among women attending antenatal care at Gondar town public health facilities, Northwest Ethiopia. BMC Pregnancy Childbirth.

[50] Seyoum B (1999). Prevalence of gestational diabetes mellitus in rural pregnant mothers in northern Ethiopia. Diabetes Res Clin Pract.

[51] Wakwoya E (2018). Gestational diabetes mellitus is a risk for macrosomia: case–control study in Eastern Ethiopia. bioRxiv.

[52] World Health Organization. Definition, Diagnosis and Classification of Diabetes Mellitus and its Complications. Part 1: Diagnosis and Classification of Diabetes Mellitus. WHO/NMH/MND/13.2 ed. Geneva: World Health Organization; 2006.

[53] Lendoye E (2022). Prevalence and factors associated to gestational diabetes mellitus among pregnant women in Libreville: a cross-sectional study. Pan Afr Med J.

[54] Frank K, Daniel K, Pidson T, Leticia K, Raymond A, Rogers K, Ritah K (2020). “Frequency and factors associated with hyperglycaemia first detected during pregnancy at Itojo General Hospital, South Western Uganda: a cross-sectional study. J Diabetes Res.

[55] Lee KW, Ching SM, Ramachandran V (2018). Prevalence and risk factors of gestational diabetes mellitus in Asia: a systematic review and meta-analysis. BMC Pregnancy Childb.

[56] Nguyen C, Pham N, Binns C, Van Duong D, Lee A. Prevalence of gestational diabetes mellitus in eastern and southeastern Asia: a systematic review and meta-analysis. J Diabetes Res. 2018. Article ID 6536974. 10.1155/2018/6536974 PMC5838488 29675432

[57] Paulo MS, Abdo NM, Bettencourt-Silva R, Al-Rifai RH (2021). Gestational diabetes mellitus in Europe: a systematic review and meta-analysis of prevalence studies. Front Endocrinol (Lausanne).

[58] Boadu WIO, Kugblenu P, Senu E, Opoku S, Anto EO (2022). Prevalence and risk factors associated with gestational diabetes mellitus among pregnant women: a cross-sectional study in Ghana. Front Clin Diabetes Healthc.

[59] Mazumder T, Akter E, Rahman SM, Islam MT, Talukder MR (2022). Prevalence and risk factors of gestational diabetes mellitus in Bangladesh: findings from demographic health survey 2017–2018. Int J Environ Res Public Health.

[60] Larrabure-Torrealva GT, Martinez S, Luque-Fernandez MA (2018). Prevalence and risk factors of gestational diabetes mellitus: findings from a universal screening feasibility program in Lima. Peru BMC Pregnancy Childb.

[61] Arora Geeti P (2015). Prevalence and risk factors of gestational diabetes in Punjab, North India: results from a population screening program. Eur J Endocrinol.

[62] Mdoe MB, Kibusi SM, Munyogwa MJ (2021). Prevalence and predictors of gestational diabetes mellitus among pregnant women attending antenatal clinic in Dodoma region, Tanzania: an analytical cross-sectional study. BMJ Nutr Prevent Health.

[63] Alharbi T, Albogami A, Alluhaidan A, Alfawaz S, Murad S (2021). Prevalence of gestational diabetes mellitus and associated risk factors among pregnant women attending antenatal care in primary health care centers in Riyadh, Saudi Arabia. J Family Med Prim Care Open Acc.

[64] Thomas OE (2018). Prevalence and risk factors of gestational diabetes mellitus in a population of pregnant women attending three health facilities in Limbe, Cameroon: a cross-sectional study. Pan Afr Med J.

[65] Mwanri AW, Kinabo J, Ramaiya K, Feskens EJ (2015). Gestational diabetes mellitus in sub-Saharan Africa: systematic review and metaregression on prevalence and risk factors. Trop Med Int Health.

[66] Wokoma FS, Celestine TJ, Enyindah CE (2001). prevalence of gestational diabetes mellitus and the pattern, behaviour, level of care and outcome of GDM pregnancies in a Nigerian antenatal population. Trop J Obstet Gynaecol.

[67] Pastakia SD, Njuguna B, Onyango BA (2017). Prevalence of gestational diabetes mellitus based on various screening strategies in western Kenya: a prospective comparison of point of care diagnostic methods. BMC Pregnancy Childb.

[68] Niyibizi J, Safari F, Ahishakiye J, Habimana J, Mapira H, Mutuku N (2016). Gestational diabetes mellitus and its associated risk factors in pregnant women at selected health facilities in Kigali City, Rwanda. J Diabetes Mell.

[69] Eades Claire E, Cameron Dawn M, Evans Josie MM (2017). Prevalence of gestational diabetes mellitus in Europe: a meta-analysis. Diabetes Res Clin Pract.

[70] Casagrande SS, Linder B, Cowie CC (2018). Prevalence of gestational diabetes and subsequent Type 2 diabetes among US women. Diabetes Res Clin Pract.

[71] Mghanga FP, Maduhu EA, Nyawale HA (2020). Prevalence and associated factors of gestational diabetes mellitus among rural pregnant women in southern Tanzania. Ghana Med J.

[72] Ali AD, Mehrass AA, Al-Adhroey A, Al-Shammakh A, Amran A (2016). Prevalence and risk factors of gestational diabetes mellitus in Yemen. Int J Womens Health.

[73] Karaçam Z, Çelİk D (2021). The prevalence and risk factors of gestational diabetes mellitus in Turkey: a systematic review and meta-analysis. J Mater Fetal Neonatal Med.

[74] dos Santos PA (2020). Gestational diabetes in the population served by Brazilian public health care. Prevalence and risk factors. RBGO Gynecol Obstet.

[75] Mishra S (2020). Risk factors for gestational diabetes mellitus: a prospective case–control study from coastal Karnataka. Clin Epidemiol Global Health (CEGH) J.

[76] Liu Z, Ao D, Yang H (2014). Gestational weight gain and risk of gestational diabetes mellitus among Chinese women. Chin Med J.

[77] Yaping X, Chunhong L, Huifen Z (2022). Risk factors associated with gestational diabetes mellitus: a retrospective case–control study. Int J Diabetes Dev Ctries.

[78] Sanabria-Martinez G, Garcia-Hermoso A, Poyatos-Leon R, Gonzalez-Garcia A, Sanchez-Lopez M, Martinez-Vizcaino V (2016). Effects of exercise-based interventions on neonatal outcomes: a meta-analysis of randomized controlled trials. Am J Health Promot.

[79] Royal College of Obstetricians and Gynaecologists. Exercise in Pregnancy. London: Statement No. 2006:4.

[80] Rönö K, Masalin S, Kautiainen H, Gissler M, Eriksson JG, Laine MK (2020). The impact of educational attainment on the occurrence of gestational diabetes mellitus in two successive pregnancies of Finnish primiparous women: a population-based cohort study. Acta Diabetol.

[81] Wong VW, Chong S, Chenn R, Jalaludin B (2019). Factors predicting recurrence of gestational diabetes in a high-risk multi-ethnic population. Aust N Z J Obstet Gynaecol.

[82] Bernstein J, Lee-Parritz A, Quinn E, Ameli O, Craig M, Heeren T, Iverson R, Jack B, McCloskey L (2019). After gestational diabetes: impact of pregnancy interval on recurrence and type 2 diabetes. BioRes Open Access.

