# Supplementary material for: Gestational diabetes mellitus and its associated factors in Ethiopia: a systematic review and meta-analysis
Source: Eur J Med Res. 2023 Mar 15;28:125. doi: 10.1186/s40001-023-01088-5 (PMC10015783; doi:10.1186/s40001-023-01088-5)
Supplement: Supplementary file 2 — Additional file 2. Quality assessment of included studies using the Joanna Briggs Institute criteria’s for assessing quality of primary studies and JBI Critical Appraisal Checklist for Studies Reporting Prevalence Data, 2019. [file 40001_2023_1088_MOESM2_ESM.docx]

Table 1: quality assessment of included studies using the Joanna Briggs Institute criteria’s for assessing quality of primary studies, 2019

| Study(Author) | Q1 | Q2 | Q3 | Q4 | Q5 | Q6 | Q7 | Q8 | Q9 | Score % |
| --- | --- | --- | --- | --- | --- | --- | --- | --- | --- | --- |
| Larebo Y et al | Y | Y | Y | Y | N | Y | N | Y | Y | 77.78 |
| Ewnetu S et al | Y | Y | N | Y | N | Y | N | Y | Y | 66.67 |
| Nigatu B et al | Y | Y | Y | Y | Y | Y | Y | N | Y | 88.89 |
| Seyoum B et al | Y | Y | Y | Y | Y | Y | Y | N | Y | 88.89 |
| Dedecha W | Y | Y | N | Y | Y | Y | N | Y | Y | 77.78 |
| Muche A et al | Y | Y | Y | Y | Y | N | Y | Y | Y | 88.89 |
| Wolka E et al | Y | Y | Y | Y | Y | N | Y | N | Y | 77.78 |
| Atlaw D et al | Y | Y | Y | Y | Y | Y | Y | N | Y | 88.89 |
| Wakwoya E et al | Y | Y | N | Y | Y | Y | Y | Y | Y | 88.89 |
| Boda B et al | Y | Y | Y | Y | Y | Y | Y | N | Y | 88.89 |
| Note:  Y - Yes, N - No, U – Unclear, NA- Not applicable  Q1= Was the sample frame appropriate to address the target population?  Q2= Were study participants sampled in an appropriate way?  Q3= Was the sample size adequate?  Q4= Were the study subjects and the setting described in detail?  Q5= Was the data analysis conducted with sufficient coverage of the identified sample?  Q6= Were valid methods used for the identification of the condition?  Q7= Was the condition measured in a standard, reliable way for all participants?  Q8= Was there appropriate statistical analysis?  Q9= Was the response rate adequate, and if not, was the low response rate managed appropriately? | | | | | | | | | |  |

**JBI Critical Appraisal Checklist for Studies Reporting Prevalence Data**

Reviewer Date

Author Year Record Number

|  | Yes | No | Unclear | Not applicable |
| --- | --- | --- | --- | --- |
| 1. Was the sample frame appropriate to address the target population? | □ | □ | □ | □ |
| 1. Were study participants sampled in an appropriate way? | □ | □ | □ | □ |
| 1. Was the sample size adequate? | □ | □ | □ | □ |
| 1. Were the study subjects and the setting described in detail? | □ | □ | □ | □ |
| 1. Was the data analysis conducted with sufficient coverage of the identified sample? | □ | □ | □ | □ |
| 1. Were valid methods used for the identification of the condition? | □ | □ | □ | □ |
| 1. Was the condition measured in a standard, reliable way for all participants? | □ | □ | □ | □ |
| 1. Was there appropriate statistical analysis? | □ | □ | □ | □ |
| 1. Was the response rate adequate, and if not, was the low response rate managed appropriately? | □ | □ | □ | □ |

Overall appraisal: Include □ Exclude □ Seek further info □

Comments (Including reason for exclusion)
